# A review of research on the intersection between breast cancer and cardiovascular research in the Women’s Health Initiative (WHI)

**DOI:** 10.3389/fonc.2022.1039246

**Published:** 2023-03-21

**Authors:** Sreejata Raychaudhuri, Christina M. Dieli-Conwright, Richard K. Cheng, Ana Barac, Kerryn W. Reding, Alexi Vasbinder, Katherine L. Cook, Vidhya Nair, Pinkal Desai, Michael S. Simon

**Affiliations:** ^1^ Department of Oncology, Hillman Cancer Center, University of Pittsburgh Medical Center, Pittsburgh, PA, United States; ^2^ Dana-Faber Cancer Institute and Harvard Medical School, Boston, MA, United States; ^3^ Division of Cardiology, University of Washington, Seattle, WA, United States; ^4^ MedStar Heart and Vascular Institute, Georgetown University, Washington, DC, United States; ^5^ Department of Biobehavioral Nursing and Health Informatics, School of Nursing, University of Washington, Seattle, WA, United States; ^6^ Division of Cardiovascular Medicine, Department of Internal Medicine, University of Michigan, Ann Arbor, MI, United States; ^7^ Department of Surgery, Wake Forest University School of Medicine, Winston-Salem, NC, United States; ^8^ Department of Hematology/Oncology, Ascension Providence Hospital/Michigan State University College of Human Medicine, Southfield, MI, United States; ^9^ Department of Oncology, Weill Cornell Medical College, New York, NY, United States; ^10^ Department of Oncology, Karmanos Cancer Institute at Wayne State University, Detroit, MI, United States; ^11^ Population Studies and Disparities Research Program, Karmanos Cancer Institute, Detroit, MI, United States

**Keywords:** breast cancer, cardiovascular disease, cancer treatment, risk factors, cancer survivors

## Abstract

Both obesity and metabolic syndrome are linked to increased incidence of type 2 diabetes, cardiovascular disease (CVD), and cancers of the breast (post-menopausal), and other obesity-related cancers. Over the past 50 years, the worldwide prevalence of obesity and metabolic syndrome has increased, with a concomitant higher incidence of associated co-morbidities and mortality. The precise mechanism linking metabolic syndrome to increased cancer incidence is incompletely understood, however, individual components of metabolic syndrome have been linked to increased breast cancer incidence and worse survival. There is a bidirectional relationship between the risk of CVD and cancer due to a high burden of shared risk factors and higher rates of CVD among cancer survivors, which may be impacted by the pro-inflammatory microenvironment associated with metabolic syndrome and cancer-directed therapies. The Women’s Health Initiative (WHI) is an excellent resource to study a dual relationship between cancer and CVD (cardio-oncology) with extensive information on risk factors and long-term outcomes. The purpose of this review is to provide an overview of research on cardio-oncology conducted utilizing WHI data with focus on studies evaluating both breast cancer and CVD including shared risk factors and outcomes after cancer. The review also includes results on other obesity related cancers which were included in the analyses of breast cancer, articles looking at cancer after heart disease (reverse cardio-oncology) and the role of Clonal Hematopoiesis of Indeterminate Potential (CHIP) as a shared risk factor between CVD and cancer. A summary of pertinent WHI literature helps to delineate the direction of future research evaluating the relationship between CVD and other cancer sites, and provides information on the opportunity for other novel analyses within the WHI.

## Introduction

Over the past 50 years, obesity has increased in prevalence, with consequent increases in morbidity and mortality (1, 2). From 2017-2018, the prevalence of obesity in the United States was estimated at approximately 42% (2) with a projected increase to above 50% after 2030 (3). In addition, over the last decade, metabolic syndrome (MS), defined by the presence of at least three out of five cardiometabolic abnormalities [high waist circumference (WC), triglycerides, blood pressure, fasting blood glucose, and low high-density lipoprotein cholesterol (HDL-C)], has also increased in prevalence (4).

Both obesity and MS have been linked to increased incidence of type 2 diabetes, cardiovascular disease (CVD), and cancers of the breast (post-menopausal), endometrium, adenocarcinoma of the esophagus, kidney, liver, gallbladder, pancreas, ovaries, small intestine, thyroid, stomach, multiple myeloma and non-Hodgkin’s lymphoma (5–9). The precise pathophysiology driving the increased incidence of cancer is incompletely understood but proposed mechanisms include shared predisposing factors such as sedentary lifestyle, and lower quality diet, or common cellular pathways related to systemic inflammation (10). Individual components of MS have been linked to higher breast cancer (BC) incidence, and worse survival among cancer survivors (11, 12). There is a proposed bidirectional relationship between risk of CVD and cancer with shared risk factors and higher rates of CVD among cancer survivors, which may be worsened by a pro-inflammatory microenvironment (10) as well as cardiotoxic cancer therapies (13).

In this review, we provide a summary of published studies within the Women’s Health Initiative (WHI) which focus on the area of “cardio-oncology” defined as intersection between cancer and CVD. The review focuses on the relationship between BC and CVD and includes studies evaluating shared risk factors and outcomes after cancer as well as “reverse cardio-oncology” investigating the risk of cancer among women with CVD. The review also covers the role of Clonal Hematopoiesis of Indeterminate Potential (CHIP) and risk of subsequent cancer (8, 12, 14–67). A PubMed search of WHI articles related to CVD and cancer, as well as other non-indexed articles were selected for the review using keywords including BC, cardio-oncology, CHIP, and WHI. When applicable, results for other obesity related cancers reported in the studies evaluating BC are also included.

The WHI includes an observational study (OS) and 3 clinical trials (CT) including the dietary modification trial (DM), the hormone therapy trial (HT) and the Calcium/Vitamin D trial (CaD). Participants could be included in one or more CT. Women were included in the OS if they were not eligible or not interested in participating in a CT. The WHI study included 161,808 postmenopausal women, aged 50-79 at enrollment, and as part of the protocol, detailed information on CVD, cancer risk factors and long-term outcomes were collected (68, 69). Participants were recruited from one of 40 U.S. clinical centers between October 1, 1993, and December 31, 1998 and had a predicted survival of at least 3 years at enrollment. Follow-up was initially through March 2005, followed by two 5-year extension periods and currently ongoing through 2027 (68, 69). The review includes publications inclusive of the entire cohort, the OS, CT or from smaller groups of participants included in ancillary studies which collected biologic or clinical information which was not part of the original protocol.

## A. Shared risk-factors

Several predisposing risk-factors and/or protective factors have been linked to CVD and cancer including physical activity, obesity, body composition, hypertension, diet, lipids, circulating cytokines and insulin resistance (70, 71). **Table 1** includes WHI studies which address shared risk factors.

**Table 1:** Summary of WHI publications on shared risk factors between cardiovascular disease and cancer, with a focus on breast cancer.

Years of study, reference	Study population/design	Main outcome	Study measure	HR, 95% CI	Main conclusion
1994-1998; Manson et al. (14)	N=73,743 WHI-OS Age= 50-79y Follow-up=5.9y	Newly diagnosed heart disease (nonfatal MI, death from coronary causes) and total cardiovascular events (MI, death from coronary causes, coronary or carotid revascularization, angina, CHF, stroke)	Quintile of total MET hr/wk (Total exercise) 1 (lowest) 2 3 4 5 (highest)	Multivariate RR of total CVD Ref 0.89 (0.75-1.04) 0.81 (0.68-0.97) 0.78 (0.66-0.93) 0.72 (0.59-0.87) P_trend_ <0.001	Walking and vigorous exercise reduce incidence of CVD events, prolonged sitting increased CVD risk
1993-1998; McTiernan et al. (15)	N=74,171 WHI-OS Age=50-79y Follow-up=4.7y	Incident invasive and *in-situ* breast cancer	Strenuous physical activity Age 18y No Yes Age 35y No Yes Age 50y No Yes	Multivariate RR of breast cancer Ref 0.94 (0.85-1.04) Ref 0.86 (0.78-0.95) Ref 0.92 (0.83-1.01)	Increased physical activity associated with reduced breast cancer risk
1993-1998; Morimoto et al. (16)	N=85,917 WHI-OS Age=50-79y Follow-up=34.8mo	Relationship between several anthropometric measures and post-menopausal breast cancer risk	Baseline BMI (kg/m^2^) in HRT never users ≤22.6 >22.6-24.9 >24.9-27.4 >27.4-31.1 >31.1	Multivariate RR for breast cancer Ref 1.52 (0.95-2.42) 1.41 (0.87-2.23) 1.70 (1.08-2.68) 2.52 (1.62-3.93) P_trend_ <0.001	Generalized obesity is risk factor for breast cancer among HRT never users; waist-to-hip ratio not associated with breast cancer risk
1994-1998; Pradhan et al. (17)	N=75,343 WHI-OS Age=50-79y Follow-up=2.9y Design= Prospective, nested, case-control study	Incidence of first MI or death from CHD	Baseline plasma concentration quartiles for: CRP 1 2 3 4 IL-6 1 2 3 4	Adjusted OR for CHD Ref 1.4 (0.8-2.8) 1.4 (0.7-2.6) 2.1 (1.1-4.1) P_trend_ = 0.046 Ref 1.7 (0.9-3.2) 1.8 (0.9-3.5) 2.1 (1.1-4.0) P_trend_ = 0.05	CRP and IL-6 independently predict CVD events, HRT increases CRP
9/1/1994-12/31/1998; Margolis et al. (18)	N=72,242 WHI-OS Age=50-79y Follow-up=6.1y	Incident fatal CHD, nonfatal MI, stroke, and total mortality	WBC count (x10^9^/L) quartiles for: Total CVD Q1 (2.5-4.7) Q2 (4.7-5.6) Q3 (5.61-6.7) Q4 (6.71-15) Total mortality Q1 Q2 Q3 Q4	Multivariate HR Ref 1.01(0.86-1.19) 1.12 (0.95-1.31) 1.47 (1.26-1.72) Ref 1.0 (0.87-1.16) 1.02 (0.89-1.19) 1.52 (1.33-1.74) P_trend_ <0.001 for all	WBC count is an independent predictor of CVD events and all-cause mortality
1993-1998; Cauley et al. (19)	N=156,351 WHI-OS and WHI-CT (all 4) Age=50-79y Follow-up=6.7y	Incident breast cancer per 1000 person-yrs	Statin use No Yes	Multivariate HR Ref 0.91 (0.8-1.05)	Overall statin use not associated with invasive breast cancer incidence
10/1993-12/1998; Chlebowski et al. (20)	N=2,996 WHI-OS and WHI-CT (all 4) Age=50-79y	Fasting insulin levels	BMI <25 25-29 ≥30 Total Physical activity (kcal/wk/kg) 0 >0-3.75 >3.75-8.75 >8.75-17.5 >17.5	Mean (SD) 8.10 (4.14) 10.4 (6.93) 14.45 (7.49) 13.03 (9.9) 11.94 (6.05) 11.33 (6.64) 10.56 (5.69) 9.48 (5.31) p<0.0001 for all	Lower BMI, higher physical activity, lower caloric intake associated with lower mean fasting insulin levels, which is a potential mediator of breast cancer risk
1993-1998; Howard et al. (21)	N=48,835 WHI-CT (DM) Age=50-79y Follow-up=8.1y Design= Interventional (reduce total fat to 20%; vegetables/fruits 5 servings/d; grains 6 servings/d)	Fatal and nonfatal CHD and stroke, and CVD (composite of CHD and stroke)	Composite CHD Stroke Total CVD	Adjusted HR 0.97 (0.9-1.06) 1.02 (0.9-1.15) 0.98 (0.92-1.05)	Dietary intervention did not significantly reduce risk of CHD, stroke or CVD
1993-2005; Prentice et al. (22)	N=48,835 WHI-CT (DM) Age=50 = 79y Follow-up=8.1y Design=Randomized, controlled, primary intervention (same as above)	Invasive breast cancer incidence	Breast cancer Incidence Mortality	Multivariate HR 0.91 (0.83-1.01) 0.77 (0.48-1.22)	Low fat diet did not result in statistically significant reduction in invasive breast cancer risk
1993-1998; Gunter et al. (23)	N=93,676 WHI-OS Age=50-79y Follow-up=77mo	Incident breast cancer	Nonusers of HT Insulin (μIU/ml) Quartile 1 (<3.9) Quartile 2 (3.9-<5.6) Quartile 3 (5.6-<8.8) Quartile 4 (≥8.8)	Multivariate HR Ref 1.04 (0.59-1.84) 1.45 (0.81-2.58) 2.48 (1.38-4.47) P_trend_ <0.001	Hyperinsulinemia is an independent risk factor for breast cancer
1993-1998; Prentice et al. (24)	N=48,835 WHI-CT (DM) Age=50-79y Follow-up=8.1y Design=Interventional (same as above)	Incidence of invasive ovarian and endometrial cancer, total invasive cancer, and invasive cancer at other sites	Cancer site Ovary Endometrium Breast Colorectal All other sites Total	Multivariate HR 0.83 (0.6-1.14) 1.11 (0.88-1.4) 0.91 (0.83-1.01) 1.08 (0.9-1.29) 0.95 (0.86-1.04) 0.95 (0.89-1.01)	Low fat diet may reduce incidence of ovarian cancer
1993-1998; Freedman et al. (25)	N=603 cases, 1206 controls WHI-CT (DM) Age=50-79y Follow-up=83mo Design=Nested case-control	Fat-breast cancer association	Log total fat and log energy FR FFQ	Adjusted standardized log RR 3.32 1.24 p=0.08	Food records (FR) may be preferable to food frequency questionnaires (FFQ) to assess diet-breast cancer relationship
1993-1998; Shikany et al. (26)	N=148,767 WHI-OS and CT (all 4) Age=50-79y Follow-up=8y	Incident breast cancer	Quintiles GL (g/d) 1 2 3 4 5 GI 1 2 3 4 5 Carbohydrate (g/d) 1 2 3 4 5	Multivariate HR For total breast cancer Ref 1.05(0.94-1.16) 0.97 (0.87-1.09) 1.10 (0.97-1.25) 1.08 (0.92-1.29) P_trend_=0.27 Ref 1.02(0.93-1.13) 1.01 (0.92-1.12) 0.97 (0.88-1.07) 1.01 (0.91-1.12) P_trend_=0.74 Ref 0.94 (0.84-1.05) 0.94 (0.84-1.05) 1.00 (0.88-1.14) 0.95 (0.8-1.14) P_trend_=0.98	No association between GL, GI and carbohydrate and total breast cancer risk, with possible association between GL and *in-situ* breast cancer
1993-1998; Caan et al. (27)	N=48,835 WHI-CT (DM) Age=50-79y Follow-up=8.1y	Invasive breast cancer incidence	Intervention vs. comparison grp No hot flashes Hot flashes	Multivariate HR 0.93 (0.84-1.03) 0.65 (0.42-1.01)	Hot flashes (HF) may identify women whose risk of invasive breast cancer can be reduced by low fat diet, mainly ER/PR positive tumors
1993-1998; Kabat et al. (28)	N=4,888 WHI-OS and CT (DM, HT, CaD) Age=50-79y Follow-up=8y	Incident breast cancer	Metabolic syndrome No Yes	Multivariate HR Ref 1.12 (0.78-1.62)	Metabolic syndrome at baseline not associated with increased risk of breast cancer, some positive association in time-dependent analyses
1993-1998; Welti et al. (29)	N=80,943 WHI-OS Age=50-79y Follow-up=20y	Incidence of obesity related cancers (breast, endometrial, colorectal)	Breast cancer Stable weight Weight gain Weight loss Weight cycling	Multivariate HR Ref 1.11 (1.03-1.20) 0.90 (0.75-1.08) 1.02 (0.95-1.21)	Weight gain and weight cycling positively associated with risk of breast and endometrial cancer
1993-1998; Luo et al. (30)	N=76,628 WHI-OS Age=50-79y Follow-up=10.3y	Invasive breast cancer incidence	Smoking history in obese women Never smoker Ever smoker Former smoker Current smoker	Multivariate HR Ref 0.96 (0.84-1.10) 0.96 (0.83-1.11) 0.96 (0.69-1.34) p=0.01	Effect of smoking on breast cancer risk was modified by obesity
1993-1998; Gunter et al. (31)	N=875 case, 839 control WHI-OS Age=50-79y Follow-up=11y	Incident breast cancer	CRP (μg/ml) Non-HT users Quartile 1 Quartile 2 Quartile 3 Quartile 4	Multivariable HR Ref 1.0(0.65-1.56) 2.28 (1.36-3.81) 1.63 (0.95-2.80) P_trend_=0.10	CRP is a risk factor for postmenopausal breast cancer among HT nonusers
Hvidtfeldt et al. (33)	N=1,601 WHI-OS Age=50 = 79y	Breast cancer incidence	BMI (5U increase) Estradiol Insulin	Total effect (extra cases per 100,000 women at-risk per yr) 52 (12.1-91.3) Proportion of total effect 21% 65.8%	Relation of BMI to breast cancer was partly mediated through estradiol, and by insulin to a greater extent
1993-1998; Phipps et al. (34)	N=155,723 WHI-OS and CT (all 4) Age=50 = 79y Follow-up=7.9y	Incidence of triple negative and ER+ breast cancer	BMI (kg/m^2^) quartiles ER+ <23.75 23.75-26.89 26.9-31.04 ≥31.05 Triple negative <23.75 23.75-26.89 26.9-31.04 ≥31.05	Multivariate HR Ref 1.19 (1.05-1.35) 1.17 (1.03-1.33) 1.39 (1.22-1.58) P_trend_<0.01 Ref 0.99 (0.67-1.46) 1.21 (0.83-1.77) 1.35 (0.92-1.99) P_trend_=0.07	Triple negative and ER+ breast cancers have similar associations with BMI and physical activity
1993-1998; Prentice et al. (35)	N= 48,835 WHI-CT (DM) Age=50-79y Follow-up=16y Design= RCT (as above)	CHD and overall CVD incidence and mortality (secondary)	Cumulative CVD outcomes (intervention + post-intervention period) Composite CHD Stroke Total CVD Cumulative mortality CHD death CVD death All-cause	Multivariate HR 1.0(0.94-1.07) 1.0(0.91-1.10) 1.0(0.94-1.05) 0.99 (0.89-1.10) 0.98 (0.91-1.05) 0.99 (0.95-1.03)	Overall no difference in CHD, total CVD or total mortality in the intervention or post-intervention periods
1993-1998; Reding et al. (36)	N=56,997 WHI-OS and CT (all 4) Age=50-79y Follow-up=5.7y	Incidence of CHD, HF, or composite cardiac events (CHD and HF)	Antihypertensive medication BB ACEi/ARB ACEi/ARB + BB CCB Diuretic	Ratio of multivariate HR among cancer vs. non-cancer cohort Ref 2.25 (1.74-4.32) 1.53 (0.64-3.63) 1.41 (0.58-3.43) 1.40 (0.65-3.00)	Among cancer survivors, 2.24-fold increased risk of total cardiac events using ACEi/ARB compared to BB
1993-2010; Foraker et al. (37)	N=161,809 WHI-OS and CT (all 4) Age=50-79y Follow-up=13y	Incident CVD, cancer and cancer subtypes (lung, colorectal, breast)	Comparing lowest with highest CVH scores Incident cancer Incident CVD	Multivariate HR 1.52 (1.35-1.72) 6.83 (5.83-8.00)	Lower ideal CVH predicts increased risk of CVD (7 times) and cancer (52%)
1993-1998; Rohan et al. (38)	N=10,960 WHI-OS and CT (all 4) Age=50-79y Follow-up=12.9y	Incident breast cancer	Whole body fat mass quintiles 1 2 3 4 5	Multivariate HR Ref 1.18 (0.86-1.62) 1.57 (1.16-2.13) 1.47 (1.08-2.02) 1.88 (1.38-2.57) P_trend_ <0.0001	All baseline DXA derived body fat measures had a positive association with breast cancer risk
1993-1998; Neuhouser et al. (39)	N=67,142 WHI-CT (all 4) Age=50-79y Follow-up=13y	Incident invasive breast cancer	Obesity grade (BMI) Normal (<25) Overweight (25-<30) Grade 1 (30-<35) Grade 2 + 3 (≥35)	Multivariate HR (all invasive breast cancer) Ref 1.17 (1.06-1.29) 1.37 (1.23-1.53) 1.58 (1.40-1.79) P_trend_ <0.001	Obesity associated with increased invasive breast cancer risk, specially ER/PR+ tumors
1993-1998; Kabat et al. (40)	N=143,901 WHI-OS and CT (all 4) Age=50-79y Follow-up=12.7y	Incidence of four obesity-related cancers (breast, endometrial, colorectal, renal)	ABSI Quintiles of cancer type Breast 1 2 3 4 5 Endometrium 1 2 3 4 5	Multivariate HR Ref 1.01 (0.94-1.09) 0.98 (0.91-1.06) 1.01 (0.94-1.09) 1.04 (0.96-1.12) P_trend_=0.33 Ref 1.23 (1.02-1.48) 1.15 (0.95-1.38) 1.02 (0.83-1.23) 1.20 (0.98-1.44) P_trend_=0.40	ABSI showed no association with risk of breast/endometrial cancer and weak associations with colorectal/renal cancer than other anthropometric measures of central obesity
1993-1998; Zheng et al. (41)	N=93,676 WHI-OS and NPAAS Age=50-79y Follow-up=9/2010 for CVD/cancer and 9/2012 for diabetes	Incident CVD, invasive cancer and diabetes	Disease category Total CVD TEC AREE Total invasive cancer TEC AREE	Multivariate HR (calibrated) 1.49 (1.23-1.81) 0.83 (0.73-0.93) 1.43 (1.17-1.73) 0.84 (0.73-0.96)	Calibrated TEC was positively related and AREE inversely related to risk of total CVD, cancer (including breast) and diabetes
1993-1998; Arnold et al. (43)	N=73,913 WHI-OS Age=50-79y Follow-up=12.6y	Incident all obesity-related cancer (colorectal, liver, gallbladder, pancreas, postmenopausal breast, endometrium, ovary, kidney, thyroid)	All-obesity related cancer Overweight duration (per 10y) Obesity duration (per 10y) OWY (per 100U) OBY (per 100U)	Multivariate HR 1.06(1.06-1.09) 1.10 (1.08-1.12) 1.12 (1.09-1.15) 1.12 (1.08-1.15)	Longer duration and intensity of overweight and obesity associated with increased risk of many types of cancer, specially breast and endometrial
1993-2005; Crandall et al. (44)	N=45,663 WHI-OS Age=50-79y Follow-up=7.2y	Time to first occurrence of CHD, invasive BC, stroke, pulmonary embolism, hip fracture, colorectal, endometrial cancer, or death from any cause (Global Index Event – GIE)	GIE No vaginal estrogen Vaginal estrogen Intact uterus Hysterectomy	Multivariate HR Ref 0.76 (0.64-0.91) 0.68 (0.55-0.86) 0.94 (0.7-1.26)	Risk of CVD and cancer not elevated in vaginal estrogen users
1993-1998; Thomson et al. (45)	N=92,295 WHI-OS and CT (HT/CaD) Age=50-79y Follow-up=14.6 ± 5.6y	Incident obesity-associated cancers (breast, colorectal, endometrium, ovary, kidney, pancreas, gallbladder, esophagus)	DED quintiles for any obesity related cancer 1 2 3 4 5	Age-adjusted sub-hazard ratio Ref 1.0(0.9-1.1) 1.05 (0.99-1.1) 1.05 (0.98-1.1) 1.1 (1.03-1.2)	Higher DED associated with 10% increased risk of obesity-related cancers, including BC (6%)
1993-1998; Chlebowski et al. (47)	N=61,335 WHI-OS Age=50-79y Follow-up=11.4y	Incident invasive BC	Weight change between baseline and Year 3 Stable (<5%) Gain (≥5%) Loss (≤5%)	Multivariate HR Ref 1.02 (0.93-1.11) 0.88 (0.78-0.98)	Weight loss (≥5%) associated with lower BC risk than stable weight
1993-1998; Kabat et al. (8)	N=21,103 WHI-OS and CT (all 4) Age=50-79y Follow-up=14.7y	Incident breast, endometrial and ovarian cancer	Quartiles of serum insulin (mg/l) <33.5 33.5->51.5 51.5-<81.5 ≥81.5	Multivariate HR for Breast cancer Ref 1.07(0.9-1.28) 1.25 (1.04-1.5) 1.41 (1.16-1.72)	Serum insulin was positively associated with breast and endometrial cancer risk; but not ovarian cancer
1993-1998; Luo et al. (48)	N=58,667 WHI-OS Age=50-79y Follow-up=12y	Incident obesity-related cancers (breast, ovary, endometrium, colorectal, esophagus, kidney, liver, multiple myeloma, pancreas, stomach, thyroid)	Intentional weight loss All Breast Intentional WC loss All Breast	Multivariate HR 0.88 (0.8-0.98) 0.9 (0.79-1.03) 0.88 (0.8-0.96) 0.9 (0.8-1.01)	Intentional weight or WC loss (≥5%) from baseline to year 3 was associated with lower risk of obesity-related cancer
1993-1998; Kabat et al. (49)	N=21,000 WHI-OS and CT (all 4) Age=50-79y Follow-up=15y	Incident BC	Metabolic phenotypes in total population MHNW MUNW MHOW MUOW MHO MUO	Multivariate HR Ref 0.86 (0.51-1.38) 1.08 (0.9-1.31) 1.17 (0.93-1.47) 1.31 (1.07-1.61) 1.61 (1.34-1.94)	Obesity and metabolic dysregulation associated with BC risk, MUO with highest risk
1993-2016; Arthur et al. (50)	N=131,833 WHI-OS and CT (all 4) Age=50-79y Follow-up=16.9y	Incident invasive BC	HLI score quintiles ≤9 10-11 12-13 14-15 ≥16	Multivariate HR for all BC cases Ref 0.93 (0.87-1.0) 0.85 (0.8-0.91) 0.75 (0.7-0.81) 0.7 (0.64-0.76) P_trend_ <0.01	4% reduction in BC risk per unit increase in HLI score
1993-1998; Iyengar et al. (52)	N= 3,460 WHI-OS and CT (all 4) Age=50-79y Follow-up=16y	Incident invasive breast cancer in women with normal BMI	Whole-body fat mass (kg) by DXA ≤18.7 18.8-22.0 22.1-25.1 >25.1	Multivariate HR Ref 1.45 (0.91-2.3) 1.68 (1.06-2.64) 1.89 (1.21-2.95) P_trend_ =0.004	In women with normal BMI, higher body fat level (by DXA) associated with higher risk of invasive BC, specially ER+
1993-1998; Reding et al. (61)	N=2,272 WHI-OS and CT Age=50-79y Follow-up=7.2y	Incidence and mortality of HFpEF and HFrEF in BC survivors	Overall mortality Hospitalized HFpEF Hospitalized HFrEF	Multivariate HR 5.65 (4.11-7.76) 3.77 (2.51-5.66)	Incidence of HFpEF hospitalizations (6.68%) higher than HFrEF (3.96%) in BC survivors; HF with higher mortality risk
1993-1998; Arthur et al. (62)	N=137,283 WHI-OS and CT (all 4) Age=50-79y Follow-up=19y	Incident invasive BC	REE quintiles Ikeda method 1 2 3 4 5 Livingston method 1 2 3 4 5 Mifflin method 1 2 3 4 5	Multivariate HR Ref 1.06(0.99-1.14) 1.14 (1.06-1.23) 1.28 (1.17-1.39) 1.39 (1.23-1.57) P_trend_ <0.001 Ref 1.03(0.98-1.13) 1.14 (1.05-1.23) 1.25 (1.14-1.37) 1.37 (1.21-1.55) P_trend_ <0.001 Ref 1.16 (0.99-1.14) 1.16 (1.08-1.25) 1.26 (1.16-1.36) 1.34 (1.21-1.48) P_trend_ <0.001	Higher REE (for all 3 methods of calculation) associated with higher BC risk
1993-1998; Desai et al. (72)	N=154,587 WHI-OS and CT (all 4) Age=50-79y Follow-up=10.8y	Incident invasive BC	Statin use	Multivariate HR 0.94 (0.83-1.06)	Statins not associated with BC risk
Desai et al. (73)	N=128,675 WHI-OS and CT (all 4) Age=50-79y Follow-up=11.5y	Diagnosis of late-stage BC and BC-specific mortality	Type of statin used and late-stage BC Lipophilic (vs. none) Hydrophilic (vs. none) BC-specific mortality (statin use over time)	Multivariate HR 0.80 (0.64-0.98) 1.06 (0.70-1.59) 0.59 (0.32-1.06)	Prior statin use associated with lower BC stage at diagnosis; no significant reduction in BC-specific mortality

WHI, Women’s Health Initiative; OS, Observational Study; CT, Clinical Trial; DM, Dietary modification; MI, Myocardial infarction; MET, Metabolic equivalent; CHD, Coronary heart disease; CVD, Cardiovascular disease; CHF, Congestive heart failure; HR, Hazard ratio; CI, Confidence interval; RR, Relative risk; OR, Odds ratio; CRP, C-reactive protein; IL-6, Interleukin 6; HRT, Hormone replacement therapy; WBC, White blood cell; BMI, Body mass index; SD, Standard deviation; HT, Hormone therapy; GL, Glycemic load; GI, Glycemic index; ER/PR, Estrogen/progesterone receptor; CaD, Calcium and vitamin D supplementation; AF, Atrial fibrillation; RCT, Randomized controlled trial; HF, Heart failure; ACEi, Angiotensin converting enzyme inhibitors; ARB, Angiotensin receptor blockers; BB, Beta blockers; CCB, Calcium channel blocker; CVH, Cardiovascular health; DXA, Dual-energy Xray absorptiometry; ABSI, A body shape index; NPAAS, Nutrition and physical activity assessment study; TEC, Total energy consumption; AREE, Activity related energy expenditure; BC, breast cancer; OWY, Overweight years; OBY, Obese years; DED, Dietary energy density; WC, Waist circumference; MHNW, Metabolically healthy/normal weight; MUNW, Metabolically unhealthy/normal weight; MHOW, Metabolically healthy/overweight; MUOW, Metabolically unhealthy/overweight; MHO, Metabolically healthy/obese; MUO, Metabolically unhealthy/obese; HLI, Healthy lifestyle index; MS, Metabolic syndrome; HFpEF, Heart failure with preserved ejection fraction; REE, Resting energy expenditure.

### Physical activity

In an analysis of 73,743 women in the OS, high levels of physical activity, reported as both walking and vigorous exercise, were associated with lower incidence of CVD, irrespective of race or ethnicity, age and body mass index (BMI), with increasing quintiles of energy expenditure associated with lower risk (P_trend_ <0.001) (14). In an analysis of self-reported physical activity at age 35, and cancer risk among 74,171 women in the OS, there was a lower risk of BC for active vs. inactive women [Relative risk (RR) 0.86, 95% confidence interval (CI) 0.78-0.95] and similar trends for physical activity reported at age 18 and 50 (15). These findings were also demonstrated in a WHI analysis which showed that higher physical activity was inversely associated with all types of BC (34). As suggested by these studies, higher levels of physical activities have the potential to lower risk of both CVD and BC.

### Obesity and body composition

Obesity and body size are well-established risk-factors for CVD (74, 75) and cancers including BC (6–9). In the OS among non-hormone therapy (HT) users, women with BMI > 31.1 had a higher risk of BC (RR 2.52, 95%CI, 1.62-3.93) (16) and in another analysis, weight cycling over 4 to 6 times was associated with a higher BC risk [Hazard ratio (HR) 1.11, 95%CI 1.03-1.20] (29). Among non-HT users, the proposed mechanism for increased risk is thought to be increased peripheral conversion of androgens to estrogen by the aromatase enzyme in adipose tissue (76). Another study using data from the CT also demonstrated a significant relationship between baseline overweight/obesity and BC risk with higher risk associated with overweight/obese status compared to normal weight [HR 1.58; 95%CI 1.4-1.79] (39). Also using OS data, longer duration of being overweight was associated with a greater risk of all obesity-related cancers [Per 10-year increment HR 1.07, 95%CI 1.06-1.09], and 5% higher risk of BC (43).

Other studies have shown that both smoking and obesity are independent risk-factors for CVD (77) and cancer (7, 8, 78). In evaluating a possible synergistic effect between smoking and obesity among 76,628 women in the OS, there was a greater BC risk noted only among non-obese women (HR 1.24, 95% CI 1.05-1.47) (30) suggesting the possibility that the anti-estrogenic effects of smoking in obese women counterbalances the carcinogenic effects of tobacco (79).

It has been proposed that the obesity - cancer association may be due to the fact that adipose tissue is metabolically active, secreting cytokines and adipokines, which play a role in breast tumorigenesis (80, 81). Supportive of this hypothesis are results in the OS which demonstrated an association between higher levels of C-reactive protein (CRP) and increased BC risk among non-HT users (HR 1.67, 95%CI 1.04-2.68) (31). Similar findings, demonstrating a relationship between higher CRP and CVD risk have also been reported in the Women's Health Study (82).

Adiposity is also associated with higher levels of endogenous estrogen and insulin, both of which are known to play a role in breast tumorigenesis (23, 83). In a study of 1,601 OS women, a 5-unit increase in BMI was associated with 50 additional BC cases per 100,000 women per year, of which 65.8% was mediated by insulin and 23.8% by estrogen (33). In contrast, the use of vaginal estrogen among OS women with or without an intact uterus was not associated with greater risk of CVD, or breast cancer (44) suggesting the lack of a systemic effect of vaginally administered estrogen.

In an analysis of both anthropometric measures and physical activity in the OS and CT, women with the highest BMI quartile compared to the two lowest quartiles had a 1.35 and 1.39-fold higher risk of triple-negative-BC (TNBC) and estrogen receptor (ER)+ tumors, respectively (34).

In an attempt to develop a more valid measure of body fat distribution, a WHI study assessed the relationship between body fat distribution and central obesity (38) using baseline dual energy X-ray absorptiometry (DXA) scans. Results from this study demonstrated a positive association between central obesity and BC risk (1.5-2 fold higher), while analyses only using anthropometric measures showed no differences in risk (38). Another analysis using a body shape index (ABSI), an index hypothesized to be an improved marker of abdominal obesity, showed no association with BC risk (40).

Other studies evaluated the impact of weight change on BC risk. In one OS analysis weight loss (≥ 5%) at 3-years was associated with a significantly lower risk compared to stable weight (< 5% loss) (HR 0.88, p=0.02), and weight gain was associated with a higher risk for TNBC (HR 1.54, 95% CI 1.16-2.05) (47). Similarly, in another OS analysis, intentional weight loss (> 5%) was associated with a lower risk of 11 obesity-related cancers (including BC) compared to stable weight [HR 0.88, 95%CI 0.8-0.98] (48).

Lastly, in another analysis, both obesity and metabolically unhealthy categories were independently associated with increased BC risk, but the metabolically unhealthy obese (MUO) phenotype demonstrated the highest risk (HR 1.62, 95%CI 1.33-1.96) (49). Also an ancillary study of 3,460 women demonstrated that higher whole body fat measured by DXA, was associated with higher BC risk among women with normal BMI (HR 1.89, 95%CI 1.21-2.95) (52).

In conclusion, while obesity and body composition are known risk factors for CVD, WHI research also demonstrates the relationship between obesity, body composition and cancer risk and provides evidence that measures of body composition utilizing DXA provides a more refined method in which to investigate this relationship. In addition, the WHI biospecimen repository has enabled research further investigating the relationship between insulin, inflammatory cytokines, hormones and cancer risk.

### Hyperlipidemia

Hyperlipidemia is a known risk-factor for CVD (84), and its association with BC has also been investigated (71, 85–87). Studies in the WHI have evaluated the relationship between statin use and BC risk. In an evaluation of 156,351 women in the WHI, there was no association between statin use and BC risk overall [HR 0.91, 95% CI 0.8-1.05] however hydrophobic statins were associated with an 18% lower risk of BC [0.82, 95% CI 0.7-0.97] (19). The essentially null results were corroborated in a later follow-up analysis (72). In another study (73) lipophilic statins were associated with a reduction in diagnosis of late-stage BC (HR 0.80, 95% CI 0.64-0.98, p = 0.035) and by a marginally lower risk of breast cancer mortality (HR 0.59, 95% CI 0.32-1.06, p = 0.075). While a protective effect of statins and breast cancer risk has not been clearly demonstrated in the WHI, other ongoing research is investigating the relationship between lipid biomarkers measured at baseline and outcomes after cancer (unpublished).

### Hyperinsulinemia, insulin resistance and impaired glucose tolerance

Fasting hyperinsulinemia is a potential mediator for breast carcinogenesis (88), and insulin and insulin-like growth factor-1 (IGF-1) may synergistically increase BC risk (70, 89). In an analysis of 2,996 women in a WHI ancillary study, lower BMI (p<0.0001), higher physical activity (p<0.001) and lower caloric intake (p<0.02) were independently associated with lower mean fasting insulin levels (20). Another OS analysis among women without diabetes showed that higher fasting insulin, but not total IGF-1 was associated with a higher BC risk (HR 1.46, P_trend_=0.02) (23). Similarly, hyperglycemia resulting from impaired glucose tolerance has been shown in other non-WHI analyses to be a risk-factor for both CVD (90) and BC (91). In another WHI ancillary study of 21,103 women, higher levels of serum insulin was associated with higher BC risk (HR 1.41, P_trend_<0.0003) (8). In another overall WHI analysis there was no significant association between dietary glycemic load (GL), glycemic index (GI), or carbohydrate intake with total BC risk (26). The WHI has added to the literature on insulin resistance and impaired glucose tolerance and BC risk suggesting a relationship between diabetes and risk of BC.

### Cardiometabolic abnormalities and heart failure

Metabolic Syndrome (MS) has been shown by others to be associated with higher risk of type 2 diabetes and CVD (92). In an analysis of MS as measured at baseline among 4,888 women in the overall WHI cohort, there was no overall relationship between MS and risk of BC, however diastolic blood pressure (DBP) showed a borderline positive association among women without diabetes (28).

Hypertension is a known risk factor for CVD (86). In a study of 56,997 cancer survivors in the overall WHI, use of angiotensin-converting-enzyme inhibitors and angiotensin-receptor-blockers was associated with 2.24-fold risk of total cardiac events, and a 1.87-fold increase in heart failure (HF) risk compared to use of beta-blockers; however, these findings were only seen among women with cancer (36).

In another analysis of 2,272 women with BC hospitalized for HF, (61) the incidence of HF with preserved ejection fraction (HFpEF) was higher (6.68%) than the incidence of HF with reduced ejection fraction (HFrEF) (3.96%). Factors associated with HFpEF included prior myocardial infarction (HR 2.83), greater WC (HR 1.99) and smoking history (HR 1.65), however these variables were not associated with HFrEF. Overall mortality among BC survivors was 5.65-fold and 3.77-fold higher among women with HFpEF and HFrEF respectively, compared to those without HF. In summary, the WHI has contributed research on the relationship between CVD and various components of CVD and BC risk. In addition, WHI investigators have emphasized the importance of differentiating the specific HF phenotype (93).

### Diet

In the WHI, several measures of dietary intake have been used to investigate the relationship between diet, CVD and cancer. An investigation of 131,833 women reported a 4% reduction in BC risk per unit increase in healthy lifestyle index (HLI) scores (94) based on factors including diet and exercise (50). Another analysis (37) demonstrated that a lower cardiovascular health (CVH) score (95) was associated with a 7-fold greater risk of incident CVD, and a 52% greater risk of incident cancer, with lung cancer having the strongest association (37).

The WHI Dietary Modification (DM) CT randomly assigned 48,835 postmenopausal women to usual diet (60%) vs intervention (40%) that focused on reduction of total fat intake to 20% of energy intake, increased vegetable and fruit intake to 5-servings and grains to 6-servings/day. As measured by food frequency questionnaire (FFQ), at baseline, women consumed 32% or more of their total energy from fat (FFQ) (96, 97).

Several DM analyses evaluated the relationship between dietary intervention, CVD and incident cancer risk (21, 22, 24, 27, 35). After 8.1 years of follow-up, the dietary intervention was not associated with a reduction in CVD (21), invasive BC (22), or ovarian or endometrial cancer (24); however, risk of ovarian cancer decreased with increased duration of dietary intervention (24). In another analysis, there were no differences in CHD, total CVD, or total all-cause mortality in either the intervention or post-intervention periods after 16-years of follow-up (35). Finally, among women on a low-fat diet, baseline vasomotor symptoms, particularly hot flashes, were associated with a lower BC risk, particularly for women with ER/progesterone receptor (PR)+ tumors, thought to be due to modulation of estrogen metabolism by diet (27).

An analysis comparing two dietary instruments (4-day food records [FR] and FFQs) among women in the non-intervention DM arm, showed that the FR over the FFQ, was a preferred method of dietary assessment for all types of dietary fats (25). Another study using data from the entire WHI, demonstrated that higher dietary energy density was associated with a 10% increased risk of any obesity-related cancer among women with a normal BMI (45).

In another OS analysis, various reductions in energy consumption were associated with lower risk of major incident CVD events and cancer. Specifically, a 20% reduction in total energy consumption (TEC) was associated with one-third lower risk, 20% increase in activity-related energy expenditure (AREE) one-fourth lower risk, and simultaneous TEC and AREE, a 50% lower risk (41). Another analysis of 137,283 women demonstrated that predicted resting energy expenditure (REE) was positively associated with invasive BC risk (62).

In summary, results from the OS strongly support a relationship between fat and energy consumption and risk of CVD and cancer, including alternative measures of healthy eating and lifestyle including the HLI and CVH. These results however have not been replicated in the DM thought to be at least in part due to poor dietary compliance among participants randomized to the intervention (21, 22). The interaction between diet and other shared risk factors for CVD and cancer, including weight loss, physical activity and body composition is complex and requires further evaluation regarding synergistic relationships or whether outcomes may differ depending on timing, pre-, during or post-cancer.

## B. Shared outcomes between cancer and CVD

**Table 2** lists WHI studies on shared outcomes between CVD and cancer with a focus on BC. In the DM, low fat dietary intervention did not result in significant changes in CHD, total CVD, or all-cause mortality in the intervention, post-intervention and cumulative (intervention + post-intervention) periods (35). In an analysis of incident CVD and total and cause-specific death rates among women with and without incident BC, over 10-years post-diagnosis, there was an increase in total mortality (HR 1.20, 95%CI 1.04-1.39) for women with localized BC, aged 70-79, compared to those with no BC. While the risk for coronary heart disease was the same for women with and without BC, CVD was the leading cause of death for women with BC diagnosed between age 70-79 (42).

**Table 2:** Summary of WHI publications on shared outcomes between cardiovascular disease and cancer, focusing on breast cancer.

Years of study, reference	Study population/design	Main outcome	Study measure	HR, 95% CI	Main conclusion
1993-1998; Prentice et al. (35)	N= 48,835 WHI-CT (DM) Age=50-79y Follow-up=16y Design= RCT (as above)	CHD and overall CVD incidence and mortality (secondary)	Cumulative mortality CHD death CVD death All-cause	Multivariate HR 0.99 (0.89-1.10) 0.98 (0.91-1.05) 0.99 (0.95-1.03)	Overall no difference in CHD, total CVD or total mortality in the intervention or post-intervention periods
1993-1998; Park et al. (42)	N=101,916 WHI-OS and CT (all 4) Age=50-79y Follow-up=10.4y (with BC) vs. 15.7 (no BC)	Incident CVD events, and total and cause-specific death rates	Event (localized BC, age 70-79y) CVD events CVD death Total death	Multivariate HR 0.84 (0.7-1.00) 0.92 (0.67-1.26) 1.20 (1.04-1.39)	CVD major contributor to mortality in women 70-79y with localized breast cancer
1993-1998; Chlebowski et al. (46)	N=48,835 WHI-CT (DM) Age=50-79y Follow-up=16.1y Design=RCT	Annualized rate of death as a result of and after BC	Cumulative outcome (intervention + post-intervention period) Invasive BC incidence Death as a result of BC Death after BC	Multivariate HR 0.97 (0.9-1.04) 0.91 (0.72-1.15) 0.82 (0.7-0.96)	Low fat diet led to significantly lower death after BC
1993-1998; Simon et al. (12)	N=8,641 WHI-OS and CT (all 4) Age=50-79y Follow-up=11.3y	Mortality from BC, CVD and other-cause	Cardiometabolic abnormalities None 1-2 3-4 None 1-2 3-4 None 1-2 3-4	Multivariate HR for mortality Breast cancer Ref 1.05 (0.86-1.29) 0.97 (0.65-1.46) CVD Ref 2.06 (1.58-2.69) 3.29 (2.25-4.82) Other-cause Ref 1.39 (1.2-1.61) 1.90 (1.49-2.44) P_trend_ <0.001 (for last 2)	Cardiometabolic risk factors are associated with CVD and other-cause mortality but not BC mortality in early-stage BC
1993-1998; Chlebowski et al. (46)	N=48,835 WHI-CT (DM) Age=50-79y Follow-up=17.7y Design=RCT (same as above)	Mortality from protocol specified cancers (breast, colorectal, endometrium, ovary) – individual and composite	Death from cancer Breast All protocol-specified Death after cancer Breast All protocol-specified	Multivariate HR 0.87 (0.7-1.10) 0.94 (0.83-1.08) 0.85 (0.74-0.99) 0.95 (0.85-1.05)	Low fat diet reduced deaths after BC, but not from or after any other cancer or cancer composite
1993-1998; Chlebowski et al. (51)	N=48,835 WHI-CT (DM) Age=50-79y Follow-up=11.5y Design=RCT (as above)	BC overall survival	BC overall survival	Multivariate HR 0.78 (0.65-0.94)	BC overall survival was greater in the dietary intervention group (10y survival 82 vs. 78%)
1993-1998; Sun et al. (53)	N=156,624 WHI-OS and CT (all 4) Age=50-79y Follow-up= 2,811,187 person yrs	Mortality from all-cause, CVD and cancer	Outcome for normal weight central obesity All-cause mortality CVD mortality Cancer mortality	Multivariate HR 1.31 (1.20-1.42) 1.24 (1.05-1.46) 1.20 (1.01-1.43)	Normal weight central obesity associated with higher all-cause, CVD and cancer mortality
1993-1998; Pan et al. (54)	N=48,835 WHI-CT (DM) Age=50-79y Follow-up=19.6y Design=RCT (as above)	Dietary intervention influence on death from BC	MS score Death from BC None 1-2 3-4 Death after BC None 1-2 3-4	Multivariate HR 1.08 (0.63-1.87) 0.8 (0.62-1.02) 0.31 (0.14-.0.69) p=0.01 0.98 (0.7-1.37) 0.86 (0.74-1.01) 0.66 (0.43-1.01) p=0.16	3-4 MS components more likely to have reduction in death from BC with low fat diet
1993-2017; George et al. (55)	N=59,388 WHI-OS Age=50-79y Follow-up=18.2y	Death from all-cause, CVD, cancer, Alzheimer’s dementia and dementia not otherwise specified	HEI-2015 Quintiles All-cause death 1 2 3 4 5 Cancer death 1 2 3 4 5	Multivariate HR Ref 0.94 (0.88-1.0) 0.88 (0.83-0.94) 0.84 (0.78-0.9) 0.82 (0.76-0.87) Ref 0.92 (0.82-1.02) 0.86 (0.77-0.96) 0.86 (0.77-0.97) 0.79 (0.7-0.88)	Higher HEI-2015 scores associated with 18% lower risk of all-cause and 21% lower risk of cancer death; but not CVD deaths
1993-1998; Pan et al. (56)	N=22,837 WHI-OS and CT (all 4) Age=50-79y Follow-up=18.9y	Cancer-specific and all-cause mortality	HOMA-IR quartiles Cancer-specific 0.05-1.09 >1.09-1.77 >1.77-3.03 >3.03-402.99 All-cause 0.05-1.09 >1.09-1.77 >1.77-3.03 >3.03-402.99	Multivariate HR Ref 1.11 (0.97-1.27) 1.14 (0.98-1.31) 1.20 (1.02-1.40) P_trend_ =0.03 Ref 1.08(1.01-1.16) 1.10(1.02-1.18) 1.42 (1.32-1.53) P_trend_ <0.001	High insulin resistance associated with higher risk of cancer-specific and all-cause mortality
1993-1998; Yuan et al. (57)	N=544 WHI-OS and CT (all 4) Age=50-79y Follow-up=19.9y	Mortality after triple-negative BC (TNBC) – BC-specific and BC overall mortality	MS components BC-specific mortality None 1-2 3-4 BC-overall mortality None 1-2 3-4	Multivariate HR Ref 0.86 (0.53-1.4) 1.13 (0.5-2.55) Ref 1.41 (1.01-1.98) 2.13 (1.22-3.71) P_trend_ =0.006	TNBC with 3-4 MS components had higher BC-specific (non-significant) and overall mortality
1993-1998; Simon et al. (11)	N=12,076 WHI-OS and CT (all 4) Age=50-79y Follow-up=10y	All-cause, CVD, cancer-specific and other-cause mortality from obesity-related cancers (breast, colorectal, endometrial, kidney, pancreatic, ovarian, stomach, liver, non-Hodgkin lymphoma)	Mortality by Cardiometabolic risk factors All-cause None 1-2 3-4 Cancer-specific None 1-2 3-4 CVD None 1-2 3-4 Other-cause None 1-2 3-4	Multivariate HR Ref 1.5 (1.36-1.65) 1.99 (1.73-2.30) Ref 1.29 (1.12-1.48) 1.37 (1.10-1.72) Ref 2.52 (1.95-3.26) 4.01 (2.88-5.57) Ref 1.45 (1.23-1.70) 2.14 (1.7-2.69) P_trend_ <0.001 (for all)	Cardiometabolic risk factors before any obesity-related cancer diagnosis significantly associated with higher all-cause, cancer-specific, CVD and other cause mortality in early-stage cancer; but not BC-mortality specifically
1993-1998; Chen et al. (59)	N=96,831 WHI-OS and CT (all 4) Age=50-79y Follow-up=18.9y	Incident CVD, and all-cause and cause-specific mortality	Dietary cholesterol quartile Incident CVD Q1 Q2 Q3 Q4 Q5 Cancer mortality Q1 Q2 Q3 Q4 Q5	Multivariate HR Ref 1.04 (0.96-1.10) 1.05 (0.96-1.12) 1.10 (1.02-1.19) 1.12 (1.03-1.21) P_trend_ <0.001 Ref 0.94 (0.86-1.03) 0.98 (0.9-1.08) 1.03 (0.93-1.13) 1.03 (0.93-1.14) P_trend_ =0.16	High dietary cholesterol and egg consumption associated with higher risk of incident CVD and all-cause mortality; but not cancer mortality
1993-1998; Dieli-Conwright et al. (63)	N=161,308 WHI-OS and CT (all 4) Age=50-79y Follow-up=9.5y	BC-specific and overall mortality	Physical activity level (all women) All-cause mortality 0 >0-2.9 3-8.9 ≥9 BC mortality 0 >0-2.9 3-8.9 ≥9	Multivariate HR Ref 0.96 (0.84-1.10) 0.80 (0.72-0.90) 0.86 (0.78-0.95) P_trend_ <0.001 Ref 1.0(0.76-1.31) 0.92 (0.74-1.15) 0.85 (0.7-1.04) P_trend_ =0.09	Higher physical activity associated with lower all-cause mortality, which did not differ by cardiometabolic risk factor number in early-stage BC

WHI, Women’s Health Initiative; OS, Observational Study; CT, Clinical Trial; DM, Dietary modification; CHD, Coronary heart disease; CVD, Cardiovascular disease; HR, Hazard ratio; CI, Confidence interval; RCT, Randomized controlled trial; BC, Breast cancer; MS, Metabolic syndrome; HEI-2015, Healthy Eating Index 2015; HOMA-IR, Homeostasis model assessment of insulin resistance; TNBC, Triple-negative breast cancer; HFpEF, Heart failure with preserved ejection fraction; HFrEF, Heart failure with reduced ejection fraction.

In contrast to the findings above, showing no effect of low-fat dietary intervention on CVD and all-cause mortality in the overall DM cohort (35), *post-hoc* analyses among women with subsequent diagnosis of BC demonstrated, fewer deaths after BC among women randomized to the intervention (low fat dietary intake) (46). Consistent with this, in an analysis of overall survival among women randomized to the dietary intervention, survival among those diagnosed with BC was significantly higher in the intervention group (10-year survival of 82 vs. 78%). There were fewer deaths from BC (68 vs. 120), other cancers (36 vs. 65) and CVD (27 vs. 64) in the intervention arm which could partly explain the improved survival (51). Lastly, in an evaluation of the influence of the dietary intervention on BC mortality by MS components, only women with 3-4 MS components had a significant reduction in BC mortality in the intervention arm (HR 0.31, p=0.01), compared to those with 0 or 1-2 MS components (54). This latter result suggests that the DM intervention may be more effective among women in the highest risk group.

In a targeted analysis of 8,641 women with early-stage BC, a higher number of CM risk-factors including high waist circumference, blood pressure, cholesterol and history of type-2 diabetes, was associated with a higher risk of CVD and other-cause mortality (P_trend_<0.001) but not BC mortality (P_trend_=0.86) (12). A similar analysis on 12,076 women with early-stage obesity-related cancers (11) showed that women with 3-4 CM abnormalities (vs. none) had 1.5, 1.37, 4.0, and 2.14-fold greater risk of death from any-cause, cancer, CVD and other-causes respectively, with no specific increase in BC-specific mortality as shown in the earlier report.

In another analysis of 156,262 women in the entire cohort, those that were normal-weight, with central obesity, compared with women that were normal-weight and no central obesity, had a higher risk of mortality due to CVD (HR 1.25; 95%CI, 1.05-1.46) as well as mortality due to cancer (HR 1.20; 95%CI, 1.01-1.43) (53). These findings support non-WHI studies which have demonstrated that excessive visceral fat is a risk-factor for greater risk of CVD and cancer (98).

Other WHI analyses have looked at diet and cancer outcomes (55, 56, 59). In a study of 59,388 women in the OS, women who had higher measured Healthy Eating Index-2015 (HEI-2015) scores, reflecting more optimal diet quality, had a 21% lower risk of all-cause mortality, and an 18% lower risk of cancer mortality, but there was no association with mortality due to CVD (55). In another analysis of 22,837 women, high baseline insulin resistance, measured as higher homeostasis model assessment of insulin resistance (HOMA-IR) scores was associated with higher cancer-specific mortality (HR 1.26, P_trend_=0.003) and all-cause mortality (HR 1.63, P_trend_<0.001) (56). Lastly in a study of 96,831 women, both higher dietary cholesterol and egg intake was associated with modestly elevated risk of incident CVD, CVD mortality, and all-cause mortality, but not cancer mortality (p=0.16 and p=0.26 respectively) (59).

An analysis of 544 women with non-metastatic TNBC showed that those with a greater number of MS components had a 27% lower 10-year BC-overall survival, non-significantly higher BC-specific mortality (HR 2.05, P_trend_=0.114) and significantly higher BC-overall mortality (HR 2.13, P_trend_=0.006), likely because of reduction in other causes of death (57); while another report showed that higher physical activity was associated with lower all-cause (HR 0.86, P_trend_<0.001), but not BC-specific mortality (HR 0.85, p=0.09) (63).

In summary, WHI analyses support the notion that shared risk-factors representing lifestyle and body composition impact both cancer and CVD outcomes, largely due to risk-factor burden. It is important for investigators interested in both CVD and cancer outcomes to investigate the impact of lifestyle interventions known to modify these risk factors, which may improve outcomes from both cancer and CVD.

## C. Reverse cardio-oncology and the role of clonal hematopoiesis of indeterminate potential

While the increased risk of CVD in cancer survivors is well described for certain cancers (13), the term “reverse cardio-oncology” describes the increased risk of cancer, among individuals with CVD, compared to the general population (100). Factors linking CVD and cancer risk as addressed in the WHI (**Supplementary Table 1**) include treatment as well as pathophysiologic pathways related to inflammation, clonal hematopoiesis of indeterminate potential (CHIP), hypoxia, microRNAs, extracellular vesicles, and circulating “cardiokines” (100).

In an analysis of 93,676 women assessing the association between baseline self-reported atrial fibrillation (AF) and incident invasive breast over 15-years follow-up, there was a 19% excess risk of subsequent BC among women with AF (HR 1.19, 95%CI 1.03-1.38). While the excess BC risk was mitigated by baseline cardiac glycoside use, the use of glycosides was also independently associated with increased BC risk (HR 1.68, 95% CI1.33-2.12), but not CRC (32). In an analysis of the relationship between HF and incident cancer over 22-years follow-up, HFpEF was associated with increased total cancer incidence (HR 1.34, 95%CI 1.06-1.67), but not HFrEF (HR 0.99, 95%CI 0.74-1.34) (58). HF overall was also associated with an increased risk of obesity-related cancers but not BC specifically.

Aging is associated with acquisition of somatic mutations in the absence of neoplasia, known as clonal hematopoiesis of indeterminate potential (CHIP), which has been linked to a higher risk of cancers as well as CVD (64, 65). In the WHI, CHIP has been shown to be associated with a greater risk of leukemias, as well as solid tumor-specific mortality, but not CVD mortality post cancer diagnosis (66, 67). In an analysis of 8,709 women with data on CHIP, the prevalence of CHIP among women free of CVD and cancer was 8.7%. Further analysis of the relationship between a healthy lifestyle score (BMI, physical activity, diet and smoking) and CHIP showed that both normal BMI and never-smoking were associated with lower odds for CHIP (OR 0.71, 95%CI 0.57-0.88) (60). Since obesity is associated with both breast cancer risk and CHIP, the relationship of CHIP with breast cancer risk is of scientific interest. In fact, recent analyses of UK biobank data suggest an increased risk of breast cancer in CHIP carriers (101) and similar analyses are ongoing in the WHI with longer follow up data with more incident breast cancer cases.

In summary WHI studies demonstrate a possible relationship between pre-existing CVD and increased cancer risk. In addition, CHIP is a shared risk-factor between CVD and cancer. More importantly, several clinical associations that are seen with breast cancer are also shared with CHIP. For example, CHIP has been associated with diabetes in several cohorts and heart failure in the TOPMed consortium (that included WHI data) (102). The complex associations of CVD risk factors, CHIP and breast cancer deserve further evaluation both in terms of mediation as well as interaction together, to lead to potential worsening of outcomes. These risk factors particularly are relevant in survivorship cohorts where shared risk factors interact further with a post chemotherapy state that can impact both cardiovascular risk and CHIP penetrance.

## Future direction

This review provides an overview of published literature on shared risk-factors and outcomes between CVD and BC, highlighting a likely bidirectional risk and adding information to a recent over-arching summary of cardiovascular research in the WHI (103). The WHI findings presented here provide a unique insight into complex associations between lifestyle risk factors, CVD and BC, and long-term outcomes including CV and cancer-specific mortality. The potential clinical and public health implications of the WHI results are significant and suggest that promotion of healthy lifestyle, and behaviors in at-risk post-menopausal women, may reduce cardiovascular and cancer mortality. Importantly, this literature provides a foundation for ongoing and future research of the association between shared risk factors between CVD and cancers of other primary sites (**Supplementary Table 1**).

## Author contributions

SR and MS developed the hypothesis, rationale, helped with data gathering, analysis, writing and editing. CD-C, RC, AB, KR, AV, KC, PD and VN helped with writing and editing the manuscript. All authors contributed to the article and approved the submitted version.
