# Supplementary figures and images for: A review of research on the intersection between breast cancer and cardiovascular research in the Women’s Health Initiative (WHI)
Source: Front Oncol. 2023 Mar 21;12:1039246. doi: 10.3389/fonc.2022.1039246 (PMC10071996; doi:10.3389/fonc.2022.1039246)

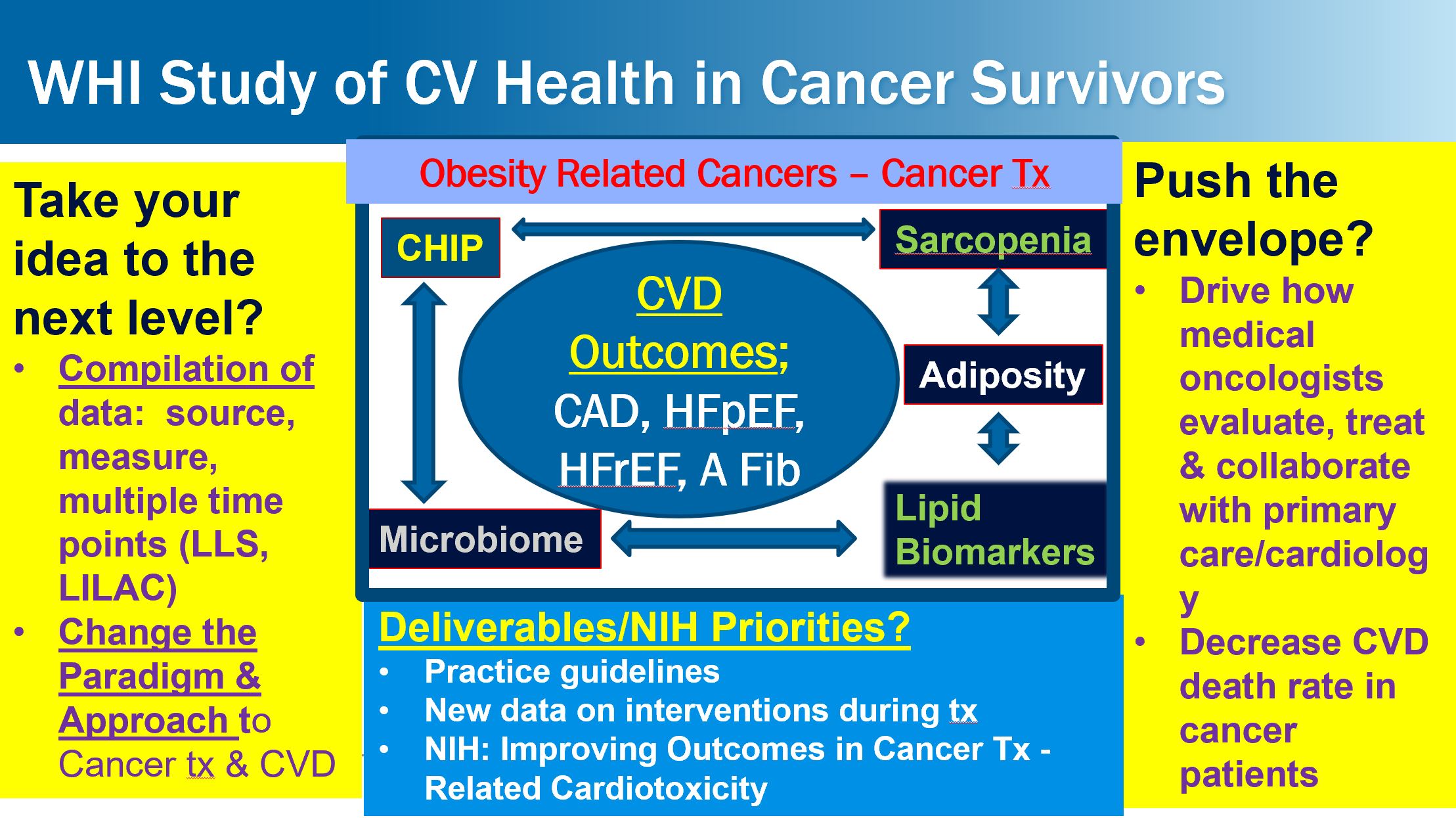

Supplement: Supplementary file 1 [file Image_1.jpeg]
