# Supplementary material for: A review of research on the intersection between breast cancer and cardiovascular research in the Women’s Health Initiative (WHI)
Source: Front Oncol. 2023 Mar 21;12:1039246. doi: 10.3389/fonc.2022.1039246 (PMC10071996; doi:10.3389/fonc.2022.1039246)
Supplement: Supplementary file 2 [file Table_1.pdf]

Supplementary Table 1: Summary of WHI publications on "Reverse cardio-oncology" and CHIP

Years of study, reference	Study population/design	Main Outcome	Study measure	HR, 95% CI	Main Conclusion
1994-1998; Wassertheil-Smoller et al (32)	N=93,676<br>WHI-OS<br>Age=50-79y<br>Follow-up=15y	Incidence of invasive breast or colorectal cancer	Self-reported history of AF at baseline<br><br>Incident breast cancer<br>Incident colorectal cancer	Multivariate HR<br><br><br><br>1.19 (1.03-1.38)<br>p=0.02<br>0.91 (0.71-1.18)<br>p=0.49	No significant association between AF and colorectal cancer, but 19% excess risk for invasive breast cancer, 68% risk with cardiac glycoside use
1993-1998; Leedy et al (58)	N=146,817<br>WHI-OS and CT (all 4)<br>Age=50-79y<br>Follow-up=22y	Incident total and site-specific cancer	Outcome<br><br>Total cancer<br>Obesity-related<br>Tobacco-related<br>Breast<br>Lung<br>Colorectal	Multivariate HR<br><br>1.28 (1.11-1.48)<br>1.24 (1.02-1.51)<br>1.24 (0.94-1.63)<br>1.17 (0.87-1.56)<br>1.58 (1.09-2.30)<br>1.52 (1.02-2.27)<br><br>P<0.001 for total cancer	HF associated with increase in incident cancer, specially lung but not breast; HFpEF associated with total cancer
1993-1998; Haring et al (60)	N=8,709<br>WHI-OS and CT (all 4)<br>Age=50-79y	Presence of CHIP	Healthy lifestyle score<br>Category 1 (0-1)<br>Category 2 (2)	Adjusted OR<br><br>Ref<br>1.13 (0.93-1.37)	Higher healthy lifestyle score was not related to CHIP; normal BMI

			Category 3 (3-4)	0.99 (0.80-1.23)<br>P_{trend} =0.95	associated with lower CHIP prevalence
Desai et al (66)	N=212<br>WHI-OS and CT (all 4)<br>Age=50-79y<br>Follow-up=9.6y	Risk of AML with pre-diagnosis somatic gene mutations	Gene mutations by age group<br><65<br><br>≥65<br><br>Total	Multivariate OR<br><br>4.39 (2.08-9.61)<br>6.19 (3.25-12.14)<br>4.86 (3.07-7.77)	Baseline mutation associated with increased odds of AML
Desai et al (67)	N=10,089<br>WHI-TOPMED consortium<br>Age=50-79y<br>Follow-up=13±6y	Incident cancer risk, all-cause, cancer-specific, CVD and other-cause mortality	Mortality<br><br><br>Cancer-specific (solid tumors)<br>CVD	Multivariate HR<br><br>1.24 (1.02-1.51)<br><br>0.63 (p=0.51)	Antecedent CHIP associated with increased solid-cancer specific mortality, but not risk of solid-tumors or CVD mortality post cancer diagnosis

Abbreviations: WHI: Women's Health Initiative; OS: Observational Study; CT: Clinical Trial; DM: Dietary modification; CHD: Coronary heart disease; CVD: Cardiovascular disease; HR: Hazard ratio; CI: Confidence interval; AF: Atrial fibrillation; HFpEF: Heart failure with preserved ejection fraction; HF: Heart failure; OR: Odds ratio; BMI: Body mass index; CHIP: Clonal hematopoiesis of indeterminate potential, TOPMED: Trans-omics for precision medicine.
